# Tetrahydroxy stilbene glycoside ameliorates neuroinflammation for Alzheimer’s disease via cGAS‐STING

**DOI:** 10.1002/alz.084633

**Published:** 2025-01-03

**Authors:** Dan Gao

**Affiliations:** ^1^ Xuanwu Hospital of Capital Medical University, Beijing, Beijing China

## Abstract

**Background:**

Alzheimer’s disease (AD), also known as senile dementia, is the most common degenerative disease of the central nervous system. Neuroinflammation is currently believed to be a crucial factor in the progression of AD, while its exact mechanism remains unclear.

**Method:**

APP/PS1 AD mice were treated with a natural active ingredient tetrahydroxy stilbene glucoside (TSG) at 40 mg/kg/day and 80 mg/kg/day respectively for 5 consecutive months, and then the Morris water maze test (MWM) and the novel object recognition test were performed to assess the effect of TSG on the cognitive and memory ability of AD mice. Moreover, central microglia activation, peripheral inflammation as well as the correlated cGAS‐STING signal pathway were analysed by Immunostaining and ELISA, which was further verified in BV2 cell experiments.

**Result:**

Treating with TSG, learning‐memory ability of AD mice was distinctly improved. Meanwhile, it was observed that the expressions of serum inflammatory cytokines and the activation of microglia in cerebral cortex and hippocampus were suppressed after TSG treatment, which was probably attributable to the decrease of cyclic GMP‐AMP synthase (cGAS) and stimulator of interferon genes (STING) triggered immune response and NLRP3 inflammasome activation. Furthermore, cell culture experiments employing LPS combined with IFN‐γ induced microglia activation showed that TSG reversed the polarization status of M1‐type microglia to restore the quiescence, and cGAS‐STING elevation was observed in the activated microglia and normalized by TSG incubation. In addition, TSG suppressed the production of inflammatory cytokines such as IL‐1β, IL‐6, TNF‐ α, IFN‐ αand IFN‐β, as well as the expression of IFN regulatory proteins such as IFIT1 and IRF7 in the LPS/IFN‐γ‐stimulated inflammatory response in BV2 cell. Finally, it was also verified that TSG are, in part, through a cGAS‐STING dependent pathway and triggered NLRP3 inflammasome activation to inhibit neuroinflammation through interfering with cGAS‐STING inhibitors.

**Conclusion:**

Our findings highlight the health benefits of TSG and its potential application in preventing cognitive disorders by inhibiting neuroinflammation through cGAS‐STING signaling pathway in AD.

